# The Intriguing mitoNEET: Functional and Spectroscopic Properties of a Unique [2Fe-2S] Cluster Coordination Geometry

**DOI:** 10.3390/molecules27238218

**Published:** 2022-11-25

**Authors:** Francesca Camponeschi, Mario Piccioli, Lucia Banci

**Affiliations:** 1 Consorzio Internuniversitario Risonanze Magnetiche Metallo Proteine, Via L. Sacconi 6, 50019 Sesto Fiorentino, Italy; 2 Magnetic Resonance Center, Via L. Sacconi 6, 50019 Sesto Fiorentino, Italy; 3 Department of Chemistry, University of Florence, Via L. Sacconi 6, 50019 Sesto Fiorentino, Italy

**Keywords:** iron-sulfur proteins, paramagnetic NMR, iron-sulfur cluster biogenesis, biophysics, human pathologies, cancer

## Abstract

Despite the number of cellular and pathological mitoNEET-related processes, very few details are known about the mechanism of action of the protein. The recently discovered existence of a link between NEET proteins and cancer pave the way to consider mitoNEET and its Fe-S clusters as suitable targets to inhibit cancer cell proliferation. Here, we will review the variety of spectroscopic techniques that have been applied to study mitoNEET in an attempt to explain the drastic difference in clusters stability and reactivity observed for the two redox states, and to elucidate the cellular function of the protein. In particular, the extensive NMR assignment and the characterization of first coordination sphere provide a molecular fingerprint helpful to assist the design of drugs able to impair cellular processes or to directly participate in redox reactions or protein–protein recognition mechanisms.

## 1. Introduction

Proteins require specific folds and three-dimensional structures in order to perform specific functions; hence, the mutation or misfolding of proteins are often associated with serious diseases. For proteins that bind cofactors, the latter play fundamental roles either in ensuring the correct three-dimensional folding of the protein, or being directly involved in the biological function of the protein itself. Fe-S clusters are ancient cofactors that are involved in a multiplicity of functions, such as electron transfer, metabolic reactions, gene expression, regulation, and DNA maintenance [1,2,3,4,5,6]. Many factors determine the wide range of specific characters taken by each Fe-S cluster: their redox potential can be tuned by the protein environment over almost 1 V range; single or multiple cluster(s) can be accommodated in a protein, either with identical or different functions; many consensus sequences are able to bind Fe-S cluster, thus providing different topologies. The functional diversity of Fe-S proteins is, therefore, strongly correlated to the properties of the cluster(s) and its neighbor region. Due to these features, Fe-S proteins have been, for four decades, a playground for bio-spectroscopists of different kinds. Recently, a novel family of [2Fe-2S] proteins, called “NEET” proteins due to the presence of the Asn-Glu-Glu-Thr (NEET) amino acid sequence at their C-termini [7], has been discovered in several organisms [8], which has been found to be involved in many processes related to normal cellular metabolism and diseases [9]. In humans, three NEET proteins are encoded by the CISD1, CISD2, and CISD3 genes. The three proteins share common structural, biochemical, and spectroscopic features, as well as similar cellular roles in the regulation of iron and ROS homeostasis in cells [9,10]. Here, we would like to review the spectroscopic properties of the first identified and most widely investigated member of the NEET family, i.e., mitoNEET, and to analyze how the unique spectroscopic properties of this protein might help to understand the role of NEET proteins in cells.

## 2. The Peculiar Properties of Human mitoNEET: A Unique Folding for a Multiplicity of Functions

**Figure 1:**
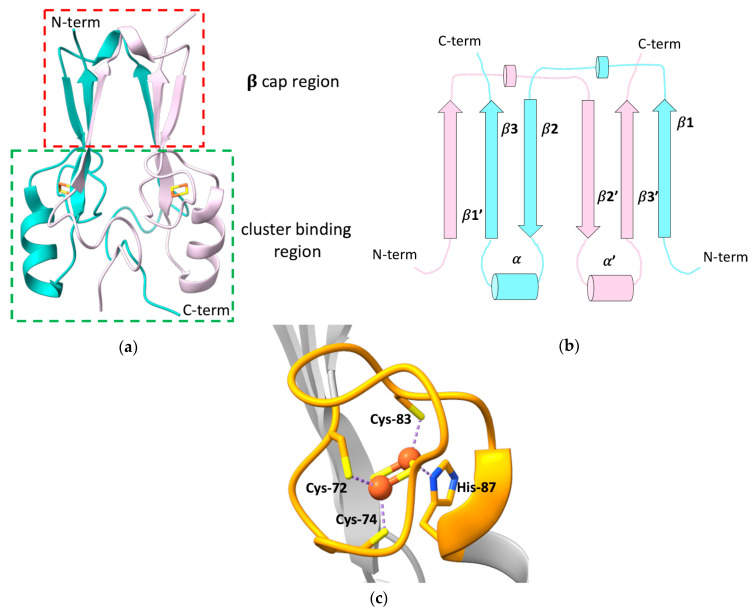
(**a**) Crystallographic structure of the soluble domain of human mitoNEET, with highlighted the β-cap and the cluster-binding regions (PDB ID: 2QH7). The two subunits forming the dimer are reported in different colors; (**b**) topology diagram illustrating the organization of the secondary structural units in the two protomers of the dimeric structure of mitoNEET; (**c**) [2Fe-2S] cluster-binding motif of human mitoNEET. The conserved CXCX_2_(S/T)X_3_PXCDG(S/A/T)H motif is highlighted in orange.

The outer mitochondrial membrane (OMM) protein mitoNEET, also known as CDGSH Fe-S domain-containing protein-1 (CISD1), is composed of 108 amino acids, encompassing a N-terminal transmembrane helix (residues 14–32) that anchors the protein to the OMM [11], and a cytosolic portion (residues 33–108) that has been widely investigated through X-ray crystallography, showing a unique, highly conserved folding [12,13,14,15]. All the crystallographic structures of mitoNEET revealed the presence of two distinct domains: a β-rich or “β-cap” region and a cluster-binding domain (Figure 1a) [12,13,15,16]. The latter contains the highly conserved CXCX_2_(S/T)X_3_PXCDG(S/A/T)H motif that binds a [2Fe-2S] cluster, and that is part of the characteristic CDGSH-type domain of 39 amino acids, which constitutes the hallmark of the “NEET” family.

mitoNEET forms an intertwined, parallel homodimer, with a pseudo two-fold symmetry [12,13,16]. Each protomer of the dimer comprises a long, flexible loop in the N-terminal part (res 33–55), whose structure has not been solved yet [14], and displays a β1-β2-α-β4 topology in the rest of the protein (Figure 1b).

In the dimer, the β-strands form two symmetric β-sheets, composed of two antiparallel β-strands from one monomer (residues 68–71 and 101–104) and an additional parallel swapped-strand from the other monomer (residues 56–61) [12,13,15]. These two β-sheets and the two loops connecting the swapped β-strands and also containing a helical turn (residues 62–64), form the so-called β-cap region [12,13,15,16], whereas the N-terminal portion of the soluble domain, the conserved α-helix formed by residues 86–94 and the loop connecting the helix to the β-cap, together with the CXCX_2_(S/T)X_3_PXCDG(S/A/T)H motif, form the cluster-binding domain [12,13,15,16]. Here, two symmetric hydrophobic cores, comprising Ile-45, Ile-56, Trp-75, Phe-80 of one monomer, and Val-98 of the second monomer [13], and two intermolecular hydrogen bonds between His-58 and Arg-73 [15], further stabilize the dimeric state of mitoNEET.

The folding of the two domains is indeed interdependent. Theoretical structure-based folding studies proposed that the rigidity in the β-cap region creates a constraint for the folding of the cluster-binding domain [16]. As part of the folding process, mitoNEET binds two [2Fe-2S]^2+/+^ clusters, one in each subunit of the dimer, using three cysteines (Cys-72, Cys-74, Cys-83) and one histidine (His-87) as ligands (Figure 1c). The His-87 ligand is located at the N-terminus of the α-helix within the cluster-binding domain and is solvent-accessible, as it is the Fe ion that it coordinates together with Cys-83. The other two ligands (Cys-72 and Cys-74) are, by contrast, buried inside the structure and bind a non-solvent-accessible Fe ion [14,15,17]. This coordination sphere, that is common to all the NEET proteins, is different from those of ferredoxin-like and Rieske proteins that coordinate [2Fe-2S]^2+/+^ clusters either with four cysteines, or with two cysteines and two histidines, respectively. The binding of the [2Fe-2S] clusters to each subunit in the dimer is responsible for the unique fold adopted by the holo protein [18], and the dimeric arrangement of the CDGSH domains seems to be essential for the stabilization of the coordination sphere of mitoNEET. Indeed it has been proposed that the β-cap domain formed by the strand swapping of the two protomers could function as an allosteric control site, modulating cluster insertion, assembly, or electron transfer [16]. Even CISD3, considered as the ancestor protein of the family, being monomeric, contains two CDGSH domains in its primary sequence and, as a consequence, two [2Fe-2S] clusters [19]. No cooperativity effect between the two clusters has been reported so far, even though inter-cluster dipolar coupling has been detected by EPR studies [20].

MitoNEET takes part into a variety of cellular processes, acting as a regulator of the homeostasis of iron and of reactive oxygen species (ROS) [21,22], as well as of the metabolism of glucose and lipids in cells, therefore modulating mitochondrial bioenergetics [21,23,24]. These functions have been linked to the observed overexpression of mitoNEET in human epithelial breast cancer cells, where the protein has been found to induce tumor cell proliferation, likely maintaining the mitochondrial functions by preventing the accumulation of iron and ROS in the mitochondrial matrix, regulating autophagy signaling, preventing autophagy [25,26,27]. Recent studies reported that mitoNEET indeed controls the formation and the integrity of inter-mitochondrial junctions and mitochondrial network morphology [24,28]. Moreover, it was observed that mitoNEET interacts with the voltage-dependent anion channel 1 (VDAC), thus regulating the free iron level within mitochondria [29]. This interaction was also proposed to be functional for the maturation of mitoNEET clusters, mediating the interaction between mitoNEET and CISD3 in a process where CISD3 transfers its [2Fe-2S] clusters from inside the mitochondria to mitoNEET [30]. In addition, mitoNEET was found to be involved in several human pathologies, such as obesity, where the overexpression of mitoNEET enhances lipid accumulation in adipocytes, but preserves insulin sensitivity [21,31,32] and neurodegeneration [33]. MitoNEET have been also linked to type 2 diabetes, since it was identified as the main cellular target of the thiazolidinedione (TZD) pioglitazone, a drug extensively used to treat insulin resistance [7], although the role of mitoNEET in the etiology of the pathology is unclear.

Despite the number of cellular and pathological mitoNEET-related processes, very few details are known about the mechanism of action of the protein in such processes. MitoNEET is supposed to play a major role in repairing the damaged [4Fe-4S] cluster on cytosolic apo aconitase IRP1 in oxidative stress conditions [18], and acts as a cluster transfer protein for several apo recipient proteins [18,34,35]. Both functions are based on a redox switch, activated by several cellular components [36,37,38,39,40]. Indeed, only [2Fe-2S]^2+^ and not [2Fe-2S]^+^ clusters can be transferred from holo mitoNEET to apo recipient proteins [18,34,35]. For this reason, the oxidized and the reduced state of mitoNEET clusters have been defined as “active” and “dormant” states, respectively [41]. The two [2Fe-2S] clusters of mitoNEET have a ∼0 mV midpoint redox potential in vitro at pH 7.5 [42,43] and in the cytoplasmic cellular environment, they are in the reduced state, as shown by EPR spectroscopy performed on *E. coli* cells containing the overexpressed cytosolic domain of human mitoNEET [44]. These findings suggest that in normal cellular conditions, mitoNEET clusters are stably bound to the protein in the reduced, dormant state. Several factors can change the redox state, and, therefore, the reactivity of mitoNEET clusters: [2Fe-2S]^2+^-mitoNEET can be reduced in vitro by biological thiols [44], reduced flavin nucleotides [36,37,38], and enzymes, such as human glutathione reductase [39]. Additionally, other Fe-S proteins, such as human anamorsin, transfer electrons to mitoNEET in vitro, showing a possible direct link between the cytosolic iron-sulfur cluster assembly (CIA) machinery and the mitoNEET cluster transfer repairing pathway [40].

Another important factor for the stability/reactivity of mitoNEET is the peculiar pH lability of its [2Fe-2S] clusters. The presence of a His residue in the first coordination sphere of mitoNEET [2Fe-2S] clusters results in a significant sensitivity to pH variations and in the pH lability of the cluster, described as a peculiar feature of the NEET proteins. Indeed, it has been proposed that the protonation of the imidazolic ring of the His-87 ligand at acidic pH facilitates the transfer of the mitoNEET [2Fe-2S]^2+^ clusters to apo recipient proteins in vitro, or their release in solution [18,34,35,41]. However, the protonation of the His-87 ligand is likely not the sole factor affecting cluster stability in mitoNEET. Indeed, Rieske proteins, which contain two His residues in their cluster coordination sphere, show a significant higher cluster stability over a wide range of pH [45]. The pH-dependent stability of mitoNEET clusters seems to be related also to a hydrogen-bonding network formed by the His-87 ligand, a conserved solvent water molecule, and the Nε of Lys-55 residue from the other polypeptide chain of the dimer [42,46,47]. Moreover, NMR and UV-visible spectroscopies showed that the stability of mitoNEET [2Fe-2S] clusters can be tuned also by the interaction with small molecules, such as the antidiabetic drug pioglitazone, which interacts with the cluster-binding region of the protein, increasing the stability of the two Fe-S clusters [15,48], or reduced nicotinamide adenine dinucleotide phosphate (NADPH), which, by contrast, destabilizes mitoNEET Fe-S clusters and induces protein unfolding [49].

For all the aforementioned characteristics, the study of the electronic and coordination structures of mitoNEET [2Fe-2S] clusters has been attracting increasing interest in the last few years, and, in addition to the above-described X-ray crystallographic studies, a variety of spectroscopic techniques have been applied to study mitoNEET in the attempt to explain the drastic difference in clusters stability and reactivity observed for the two redox states and to clarify the cellular function of the protein. The similarities and the differences between the oxidized and reduced states of the mitoNEET [2Fe-2S] clusters and those of other Fe-S proteins are crucial aspects for understanding their modes of action and their role in the physiological processes.

## 3. Spectroscopic Characterization of the Reduced and Oxidized Forms of mitoNEET

### 3.1. Electronic Spectroscopy

**Figure 2:**
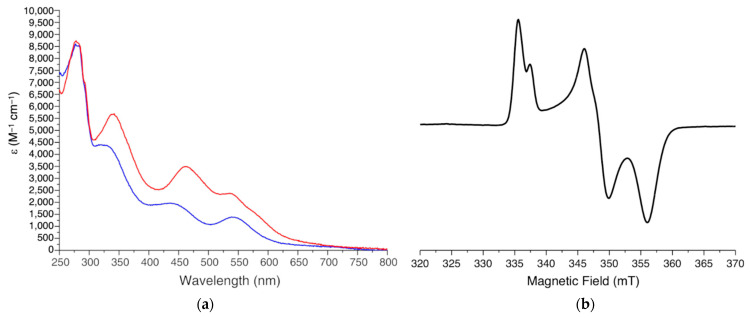
(**a**) UV-Visible spectra of oxidized [2Fe-2S]^2+^ mitoNEET (red line) and of dithionite reduced [2Fe-2S]^+^ mitoNEET (blue line). ε values are based on monomeric protein concentration. (**b**) CW X-band EPR spectrum of dithionite reduced [2Fe-2S]^+^ mitoNEET, acquired at 45 K, 1 mW.

UV-visible (UV-vis) spectroscopy has been widely used to characterize the oxidized and reduced states of the two [2Fe-2S] clusters of mitoNEET [18,50] to follow their redox properties [36,39,40], to investigate the cluster transfer activity of mitoNEET as a function of pH [34,35,41], and to characterize the system in the presence of ROS species [35,41]. Indeed, the UV-vis spectra of oxidized and reduced mitoNEET are significantly different, thus allowing the changes in the redox state of the clusters to be easily followed. The UV-vis spectrum of oxidized [2Fe-2S]^2+^ mitoNEET shows intense absorption bands at ~340 and 458 nm and bands with lower intensity between 535 and 580 nm (Figure 2a) [35,41]. This pattern resembles that of several types of [2Fe-2S] cluster-containing proteins, including all-Cys ferredoxin [51] and Rieske Fe-S proteins [52]. Therefore, as observed for Rieske proteins, the presence of a N donor atom in the first coordination sphere does not affect the optical properties of tetrahedral Fe^3+^ ions.

The absorption spectra of the two reduced [2Fe-2S]^+^ clusters of mitoNEET show a significant decrease in the intensity of all the absorption bands. This is consistent with what observed for other [2Fe-2S] cluster-containing proteins, whose spectra are generally less featured in their reduced, [2Fe-2S]^+^ state. However, reduced mitoNEET also shows an absorption peak at 540 nm, which is often observed in valence-localized, dithionite-reduced [2Fe-2S]^+^ centers [53,54] and provides spectroscopic evidence that the His ligand breaks the symmetry of the [2Fe-2S] cluster and drives the reduction of one of the two iron ions, at variance with a valence delocalized state which is present in human ferredoxins and in many proteins of the mitochondrial ISC machinery [55,56,57].

### 3.2. EPR Spectroscopy

The first continuous wave (CW) X-band EPR spectra of full-length mitoNEET were obtained on outer-mitochondrial membrane particles purified from bovine heart mitochondria [58] and showed a splitting of the rhombic EPR signal that was initially assigned to two distinct *S* = 1/2 spins of two different reduced [2Fe-2S]^+^ clusters bound to mitoNEET. The S = 1/2 ground state of the reduced [2Fe-2S]^+^ cluster arises from the antiferromagnetic coupling of the high-spin Fe^3+^ (S = 5/2) and the high-spin Fe^2+^ (S = 2) ions. Later on, EPR spectra acquired on whole *E. coli* cells overexpressing the soluble portion of mitoNEET and on several purified constructs encompassing the soluble domain of the protein [44,59] revealed that the splitting of the rhombic EPR signal resulted from the dipolar interaction of the electron spins of the two adjacent, reduced clusters in the dimeric unit of mitoNEET [20]. Specifically, the spectra of fully reduced mitoNEET show an anisotropic lineshape, corresponding to a rhombic g-tensor, with principal g values of 2.005, 1.937, and 1.895 (Figure 2b). The average value for [2Fe-2S]^+^(Cys)_3_(His)_1_ mitoNEET clusters (g_av_ = 1.945) is intermediate between the typical values of the plant ferredoxin [2Fe-2S]^+^(Cys)_4_ (g_av_ = 1.96) and Rieske [2Fe-2S]^+^(Cys)_2_(His)_2_ clusters (g_av_ = 1.91) [60].

A detailed analysis of the EPR spectra acquired on purified mitoNEET at pH 8, performed by considering spin–spin interactions between all four iron ions in reduced mitoNEET clusters [60,61], suggested, for the first time, the occurrence of valence localization, with the outermost iron ion, coordinated by His87 and Cys83 ligands, being in the ferrous state in the reduced mitoNEET [2Fe-2S]^+^ clusters [20]. This is in line with what generally reported for Rieske proteins, where the extra valence of the reduced [2Fe-2S] cluster is always localized on the Fe ions that is coordinated by the 2 His residues [62,63,64]. However, ^14^N and ^15^N ESEEM/HYSCORE experiments highlighted significant differences between mitoNEET and Rieske [2Fe-2S] proteins concerning the cluster g-tensor orientation and interaction with weakly coupled backbone amide nitrogen nuclei [20]. A lower electron spin density for the backbone amide nitrogens of mitoNEET forming hydrogen-bonds with the reduced cluster was observed with respect to that of Rieske-type proteins, reflecting their differences in the cluster structure and coordination. These observations highlighted the contribution of the interaction of the cluster with the protein environment for determining the cluster electronic structure and then possibly determining the functional properties of the protein.

Furthermore, the differences in the isotropic coupling constant of the iron-bound histidine N_δ_ suggest that small differences of iron coordination bonds and angles between mitoNEET and Rieske proteins may affect the unpaired electron spin density delocalization onto the histidine ligand [59].

### 3.3. Mössbauer Spectroscopy

Mössbauer spectra acquired on oxidized and reduced mitoNEET highlighted the high inequivalence of the two iron ions of the [2Fe-2S] clusters, in both the oxidized [2Fe-2S]^2+^ and reduced [2Fe-2S]^+^ cluster states [18,41]. In ferredoxins and ferredoxin-like proteins, the two high-spin, 2-Cys coordinated Fe^3+^ ions of the oxidized [2Fe-2S]^2+^ cluster experience nearly identical chemical environments, and the S = 0 ground state resulting from their antiferromagnetic coupling gives rise to a symmetric quadrupole doublet, with an isomer shift of δ ~ 0.27 mm/s [65]. By contrast, in mitoNEET, the two Fe^3+^ centers of the oxidized [2Fe-2S]^2+^ cluster experience a different chemical environment due to their different coordination (i.e., 2 Cys vs. 1 Cys and 1 His). As a result, Mössbauer spectra of oxidized [2Fe-2S]^2+^ mitoNEET show two distinct quadrupole doublets, corresponding to the two inequivalent Fe^3+^ centers of the cluster. Specifically, the Fe^3+^ ion coordinated by 1 Cys and 1 His residue exhibits larger ΔE_Q_ and isomer shift than that coordinated by 2 Cys [66]. The two doublets are present in a 1:1 ratio, with isomer shifts of δ ~ 0.26 and 0.30 mm/s. This behaviour is similar to that reported for the oxidized [2Fe-2S]^2+^ clusters of the scaffold protein IscU and of the transcription factor IscR in *E. coli* [18,67], both binding a [2Fe-2S]^2+^ cluster with three cysteines and one His, and having a solvent-exposed coordination site [68], as well as for the majority of the [2Fe-2S]^2+^ Rieske proteins (see Table 1) [65]. The difference in the isomer shift for the two doublets in oxidized mitoNEET spectra (0.04 mm/s) is similar to that observed for IscR [67] and for IscU (0.05 mm/s, [68]), whereas for the two iron centers of the [2Fe-2S]^2+^ clusters of Rieske proteins, which have the most different coordination sites, larger differences in isomer shifts were reported (0.08 mm/s, [66]).

In the reduced state of mitoNEET, the inequivalence of the two iron ions is enhanced by the presence of an extra valence electron that is localized on the iron ion coordinated by the His residue [20,59]. The Mössbauer spectra of this state show two quadrupolar doublets corresponding to the Fe^3+^ and Fe^2+^ ions, with significantly different isomer shift (δ ~ 0.32 mm/s and 0.68 mm/s, respectively) [44], at variance with those reported for 4Cys-coordinated, valence-delocalized [2Fe-2S]^+^ clusters, such as those of human anamorsin, whose Mössbauer spectra only show one symmetric quadrupole doublet for the two identical Fe^2.5+^ ions [55]. In mitoNEET, the different oxidation state of the two iron ions determines the properties of the Mӧssbauer spectra: indeed, the spectra are very similar to those obtained for other, reduced, valence-localized [2Fe-2S]^+^ clusters, such as those reported for 4Cys-coordinated [2Fe-2S]^+^ clusters of plant ferredoxins [69,70], for the 3Cys-1His-coordinated [2Fe-2S]^+^ cluster of *E. coli* IscR [67], and for the 2His-2Cys-coordinated cluster of *P. putida* Rieske protein [71]. However, in these systems, the isomer shift of the Fe^2+^ site increases as the number of coordinated cysteines decreases [41]: δ ~ 0.62 mm/s for *A. aeolicus* FdI (two Cys), δ ~ 0.70 mm/s for human mitoNEET or *E. coli* IscR (one Cys and one His), and δ ~ 0.75 mm/s for Rieske proteins (two His), thus emphasizing the capacity of Mossbauer spectroscopy to highlight differences among different [2Fe-2S] centers, as well as for other Fe-S clusters [65,72].

### 3.4. NMR Spectroscopy

Standard 2D ^1^H-^15^N HSQC spectra of ^15^N-labeled oxidized [2Fe-2S]^2+^ mitoNEET showed, in the pH range 7.0–8.0, well-dispersed signals, indicating the presence of well-defined secondary structure elements. About 70% of the backbone NH resonances were visible in the spectra, and ~30% NH resonances broadened beyond detection due to paramagnetic relaxation enhancement induced by the two bound [2Fe-2S]^2+^ clusters [41,75]. After reduction of the clusters with DTT or sodium dithionite, small but significant changes were observed in the 2D ^1^H-^15^N HSQC spectrum of ^15^N-labeled [2Fe-2S]^+^ mitoNEET. The observed differences are not determined by paramagnetic effects because the contribution to chemical shifts for uncoordinated residues is negligible. Small structural changes seem to affect mainly the protein regions involved in inter-subunit contacts, such as the network of interactions involving Asp-96 with Ile-45 or Phe-60 with Ile-103. Due to the paramagnetism of the clusters, standard HSQC experiments are blind in the immediate proximity of the metal center; therefore, they cannot provide information on local conformational changes in the region surrounding the Fe-S clusters. Indeed, it was reported that the reduction of mitoNEET [2Fe-2S]^2+^ clusters is coupled to the protonation of the Nε of the His-87 ligand [42,46]. This has led to the definition of the process as a proton-coupled electron transfer [42,46]. Interestingly, additional protonation events involving conserved residues belonging to the cluster-binding region of mitoNEET and forming a complex network of conserved hydrogen bonds [12,15] have been proposed to be responsible for cluster stability [13,42,46], being such hydrogen bonds responsible also for the inter-residue contacts that give rise to the typical NEET fold.

### 3.5. Paramagnetic NMR and Antiferromagnetic Coupling Properties

In Fe-S proteins, the number of iron ions, their oxidation states, and the magnetic coupling among them determine the chemical shift and the relaxation rates of the signals arising from the cluster-bound residues [76,77,78]. Paramagnetic NMR spectroscopy can provide unique and detailed information, at room temperature, on the nature of the cluster, its oxidation state, and reactivity [79,80]; it is, therefore, highly complementary to Mössbauer and/or EPR spectroscopy, which provide information at low temperatures. Indeed, room temperature paramagnetic NMR data have been extremely useful to describe in vitro mechanisms for the biogenesis and transfer of clusters, such as the formation of [4Fe-4S] clusters in the ISC and CIA machineries [81,82,83], and to monitor catalytic processes in radical SAM enzymes [84].

In the case of oxidized [2Fe-2S]^2+^ proteins, the two Fe^3+^ ions are antiferromagnetically coupled and provide a S = 0 electronic ground state, and, therefore, all [2Fe-2S]^2+^ proteins are EPR-silent. However, at room temperature, the excited levels of the electron spin ladder are populated and give rise to contact hyperfine shifts for the NMR signals of Fe-bound residues.

**Figure 3:**
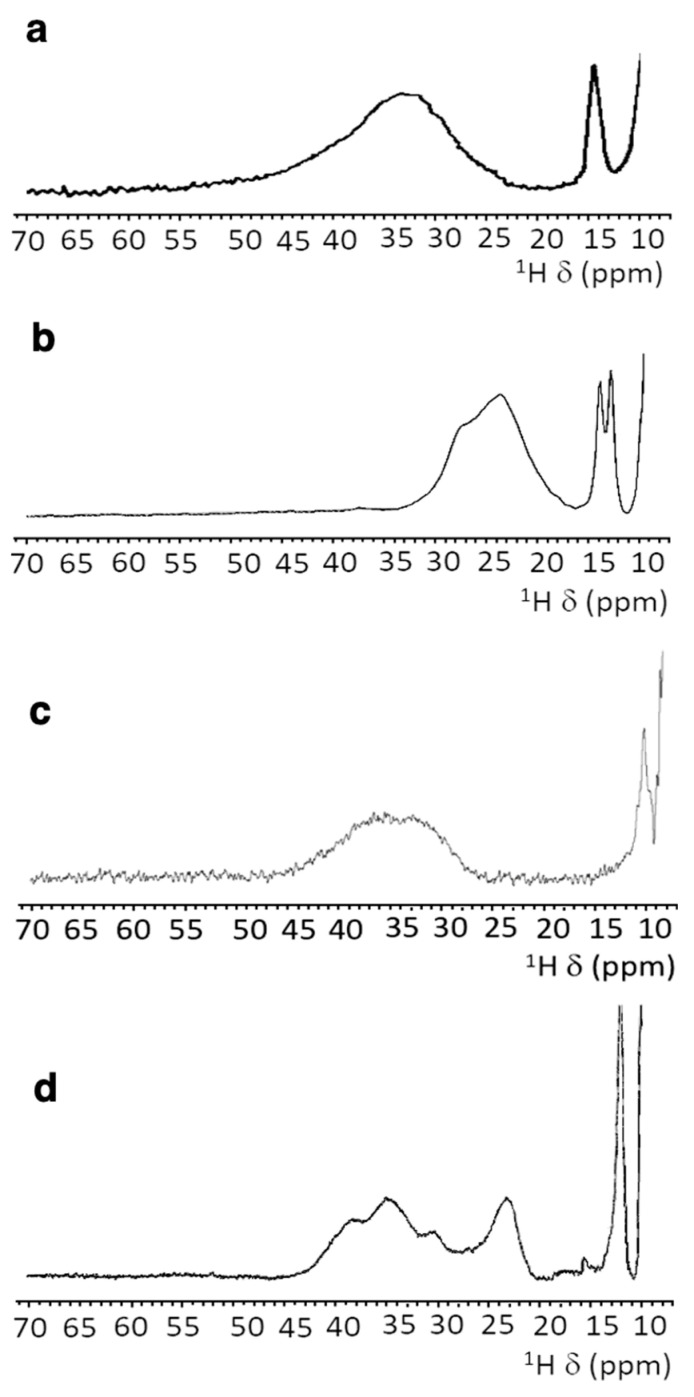
Paramagnetic ^1^H NMR spectra of [2Fe-2S]^2+^-containing proteins: (**a**) ferredoxin from red algae [85]; (**b**) human glutaredoxin-5 [56]; (**c**) human anamorsin [91]; (**d**) human ISCA2 [83].

Different [2Fe-2S]^2+^ proteins provide different NMR spectra, as summarized in Figure 3. For plant-type electron-transfer ferredoxins [85] and for the Rieske-type ferredoxin from Xanthobacter strain Py2 [86], only a very broad and unresolved feature is observed, in the 28–35 ppm range, arising from the unresolved eight cysteine βCH_2_ signals. By contrast, other [2Fe-2S]^2+^ proteins, such as vertebrate ferredoxins [87] and the human proteins ISCA1 and ISCA2 [56,83], involved in the mitochondrial ISC machinery [2,88], show a larger signal dispersion and, for human ferredoxins FDX1 and FDX2, also larger chemical shifts, up to about 45 ppm [57,89,90].

In these systems, the NMR spectra have sharper signals with respect to plant-type ferredoxins, although still insufficient to attempt individual resonance assignment for any of these systems.

**Figure 4:**
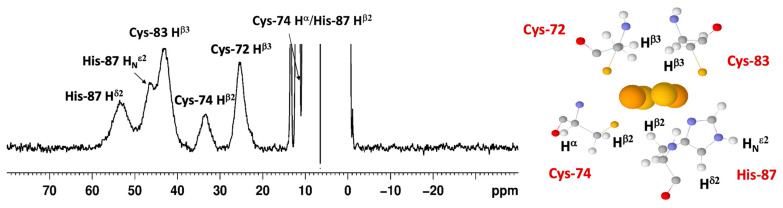
Paramagnetic ^1^H 1D spectra of oxidized [2Fe-2S]^2+^ mitoNEET at 600 MHz, 283 K [75].

In this frame, the paramagnetic NMR spectrum of oxidized mitoNEET is quite amazing and represents, to some extent, a breakthrough. As observed in Figure 4, the NMR spectrum of oxidized mitoNEET shows six well-resolved signals in a 60–10 ppm range. The observed shifts are approximately 30% larger than those of human ferredoxins, so far considered to have the most resolved spectrum of a [2Fe-2S]^2+^ protein, and about a factor of two larger than human anamorsin (Figure 3c) [91]. The signal resolution of oxidized mitoNEET is such that not only can we measure the individual relaxation properties of these signals, but we can also perform a series of 1D NOE experiments useful to identify, in the proximity of the paramagnetic center, signals of the neighbor residues [92]. This allowed us to propose a tentative assignment, that, although not supported by an unambiguous network of scalar couplings, is based on solid grounds [75].

The low symmetry of the coordination sphere partly explains the NMR behavior. It has been shown, for Anabena-7120 ferredoxin, that a Cys-to-Ser mutation increases the downfield shifts and the signal dispersion of the paramagnetic NMR spectrum [93], thus supporting the hypothesis that a low-symmetry chromophore provides better-resolved NMR spectra for the oxidized [2Fe-2S]^2+^ state of the proteins, probably because different coordination bonds affect the pattern of the spin density distribution. However, the significant line narrowing suggests a variation of the electronic structure of the cluster itself. For two equal S = 5/2 spins and *J* values lower than kT (206 cm^−1^ at 298K), the paramagnetic contribution to the nuclear relaxation of atoms around the cluster (both contact and dipolar terms) is significantly smaller than that originating from a single S = 5/2 spin of the same nature. Indeed, this is the reason why the paramagnetic NMR spectra of [2Fe-2S]^2+^ are observable, at variance with single Fe^3+^ rubredoxins. Furthermore, when J is of the order of kT (*J* values in [2Fe-2S]^2+^ proteins are in the range 180–280 cm^−1^), nuclear relaxation can be very sensitive also to small *J* variations. An increase of the J value determines a decrease of the population of the excited states and of the magnetic susceptibility of the system, thus resulting in smaller contributions to paramagnetic relaxation enhancement. Recently, a relatively similar NMR spectrum has been reported for the bacterial protein FhuF [94], in which the data of the oxidized form were rationalized with a J value of 300 cm^−1^, i.e., higher than previously reported for [2Fe-2S]^2+^ clusters. FhuF binds the [2Fe-2S]^2+^ cluster with four cysteines, but it has a unique consensus sequence and binding topology, which provides an unusually distorted geometry around the cluster.

In reduced [2Fe-2S]^+^ proteins, the 1D paramagnetic NMR spectra have been correlated with the different electronic properties of the cluster: when the extra electron is mainly localized on one individual iron ion, relatively sharp and well-separated NMR signals for all βCH_2_ and αCH cysteine protons are observed [95]. When valence is delocalized on the two iron ions, much broader lines, often undetectable, occur for ^1^H signals [90]. The NMR spectra of reduced [2Fe-2S]^+^ Rieske proteins show relatively sharp and well-resolved NMR signals over a 100–20 ppm range and, therefore, are consistent with the valence localized model. Puzzling enough, no hyperfine shifted signals are detected for reduced [2Fe-2S]^+^ mitoNEET. The hypothesis that the electron distribution within the cluster is different from Rieske proteins seems unlikely: the presence of a histidine in the coordination sphere and the structural asymmetry of the cluster in mitoNEET, with one iron ion exposed to the surface and the other buried inside the protein, are supposed to drive the reduced form of mitoNEET towards a fully localized valence, with the extra electron located on the surface-exposed iron ion. Other spectroscopies support this view, as already pointed out [20,44,59].

A possible rationale for the absence of observable signals in the paramagnetic ^1^H NMR spectrum of mitoNEET is the occurrence of dynamics/conformational phenomena that determine additional line broadening to the signals of the cluster-bound residues and eventually prevent their observation. Actually, Rieske proteins and plant-type ferredoxins (which share the same NMR features in their reduced states) are electron transfer proteins, whereas mitoNEET plays a major role in restoring the Fe-S cluster on cytosolic aconitase IRP1 under oxidative stress conditions [18] and acts as a cluster transfer protein for several apo recipient proteins [18,35,36]. These functions are based on a redox switch that is activated by several cellular cofactors [36,38,39,40,44]. It is, therefore, possible that, in order to perform its function, mitoNEET switches between different conformational states, with the redox state change being one of the ways of regulating these transitions. However, another structural difference between mitoNEET and Rieske proteins is the presence of two [2Fe-2S] clusters in all NEET proteins. As already shown for the two [4Fe-4S] ferredoxins [96], the reduction of a two cluster protein may give rise to a fully reduced form and also to an intermediate form with one cluster oxidized, the other reduced, and the occurrence of electron self-exchange between the two clusters. The occurrence of a slow (on the NMR time scale) exchange process between the oxidized and reduced forms and of a fast exchange process between the two clusters in the intermediate state provide a situation that has been successfully monitored by paramagnetic NMR only for the *C Pasteurianum* ferredoxin, where the small protein size and the two [4Fe-4S] ^2+/+^ cluster provides sharp and well-resolved NMR signals [96]. If a partial reduction of mitoNEET would provide an intermediate state, with one oxidized and one reduced cluster, then the much larger signal linewidths of residues bound to [2Fe-2S]^2+/+^ cluster, compared to those of ferredoxins, would prevent the identification of the intermediate species and eventually, due to the exchange, also of the fully reduced form.

## 4. Hints for Future Studies: Targeting mitoNEET to Fight Cancer

MitoNEET and the other members of the NEET proteins family are involved in numerous human pathologies and key cellular processes [9,17]. Despite the number of studies and the ensuing amount of information that has been gathered about the NEET proteins, many issues require future studies. In this respect, one of the most attractive aspects is the recently discovered existence of a link between NEET proteins and cancer. Indeed, several studies showed how different human cancer cells contains significantly increased levels of mitoNEET and NAF-1 [9], which play a critical role in promoting tumor growth and metastasis. Attempts to explain the role of NEET proteins in cancer proliferation are based on their well-documented function as regulators of mitochondrial stability and iron and ROS homeostasis in cells [25,27,97,98], their ability to protect cells from the activation of apoptosis and autophagy [25,97], as well as to protect the mitochondrial oxidative phosphorylation system (OXPHOS) proteins from oxidative stress [26]. All the aforementioned functions of the NEET proteins have been reported to rely on the presence of their peculiar Fe-S clusters. However, the overall mechanism for the function of the NEET proteins in cancer proliferation is still elusive.

This leaves room for a more detailed description and understanding of the cellular pathways in which each of the NEET proteins takes part. It will be crucial to identify the network of interactions of the NEET proteins, which is, so far, mostly unknown, and to define at the atomic level how they interact with their protein partners, and the role played by their redox- and pH-sensitive Fe-S clusters. New anticancer strategies having the NEET proteins and their Fe-S clusters as suitable targets could be designed to inhibit cancer cell proliferation.

In summary, the extensive spectroscopic characterization that has been performed so far on mitoNEET and that is reviewed in this work provides an invaluable background of information. In particular, the extensive NMR assignment and the characterization of first coordination sphere provides a molecular fingerprint that can be powerful to assist the design of anti-cancer drugs able to either stabilize or destabilize mitoNEET clusters, thus impairing cellular processes where the clusters have to be transferred from mitoNEET to apo recipient proteins, or directly participate in redox reactions or protein–protein recognition mechanisms.

## Figures and Tables

**Table 1:** Mossbauer parameters for selected [2Fe-2S]^2+^ and [2Fe-2S]^+^ proteins.

System	Cluster Type	Formal Valences	Fe Ligands	S_tot_	δ (mm/s)	|ΔEQ| (mm/s)	Refs.
**FdI *A*** **.** ***aeolicus***	[2Fe-2S]^2+^	2 Fe^3+^	4 Cys	0	0.27	0.60	[70]
**putidaredoxin *P. putida***	[2Fe-2S]^2+^	2 Fe^3+^	4 Cys	0	0.27	0.60	[69]
**yeast Grx3**	[2Fe-2S]^2+^	2 Fe^3+^	4 Cys	0	0.29	0.55–0.76	[73]
**human ISCA1**	[2Fe-2S]^2+^	2 Fe^3+^	4 Cys	0	0.28	0.50	[74]
**human ISCA2**	[2Fe-2S]^2+^	2 Fe^3+^	4 Cys	0	0.27	0.53	[74]
**anamorsin (site 1)**	[2Fe-2S]^2+^	2 Fe^3+^	4 Cys	0	0.26	0.57	[55]
**anamorsin (site 2)**	[2Fe-2S]^2+^	2 Fe^3+^	4 Cys	0	0.28	0.39	[55]
**Rieske *thermus thermophilus***	[2Fe-2S]^2+^	Fe^3+^ Fe^3+^	2 Cys 2 His	0	0.24 0.32	0.32 0.91	[66]
**mitoNEET**	[2Fe-2S]^2+^	Fe^3+^ Fe^3+^	2 Cys 1 Cys 1 His	0	0.26 0.30	0.47 0.96	[18,41]
***E. coli*** **IscU**	[2Fe-2S]^2+^	Fe^3+^ Fe^3+^	2 Cys 1 Cys 1 His	0	0.27 0.32	0.66 0.94	[68]
***E. coli*** **IscR**	[2Fe-2S]^2+^	Fe^3+^ Fe^3+^	2 Cys 1 Cys 1 His	0	0.27 0.30	0.48 0.72	[67]
**yeast Fra2-Grx3**	[2Fe-2S]^2+^	Fe^3+^ Fe^3+^	2 Cys 1 Cys 1 His	0	0.30 0.32	0.50 0.82	[73]
**FdI *A. aeolicus***	[2Fe-2S]^1+^	Fe^3+^ Fe^2+^	2 Cys 2 Cys	1/2	0.30 0.62	1.0 3.0	[70]
**putidaredoxin *P. putida***	[2Fe-2S]^1+^	Fe^3+^ Fe^2+^	2 Cys 2 Cys	1/2	0.35 0.60	0.65 2.70	[69]
**anamorsin (site 1)**	[2Fe-2S]^1+^	2 Fe^2.5+^	4 Cys	1/2	0.26	0.57	[55]
**anamorsin (site 2)**	[2Fe-2S]^1+^	2 Fe^2.5+^	4 Cys	1/2	0.28	0.39	[55]
**Rieske *thermus thermophilus***	[2Fe-2S]^1+^	Fe^3+^ Fe^2+^	2 Cys 2 His	1/2	0.31 0.74	0.63 3.05	[66]
***E. coli*** **IscR**	[2Fe-2S]^1+^	Fe^3+^ Fe^2+^	2 Cys 1 Cys 1 His	1/2	0.33 0.70	1.09 3.4	[67]
**mitoNEET**	[2Fe-2S]^1+^	Fe^3+^ Fe^2+^	2 Cys 1 Cys, 1 His	1/2	0.32 0.68	1.07 3.15	[41]

## Data Availability

Not applicable.

## References

[1] Beinert H. (2000). Iron-Sulfur Proteins: Ancient Structures, Still Full of Surprises. J. Biol. Inorg. Chem..

[2] Lill R. (2009). Function and Biogenesis of Iron-Sulphur Proteins. Nature.

[3] Rouault T.A. (2019). The Indispensable Role of Mammalian Iron Sulfur Proteins in Function and Regulation of Multiple Diverse Metabolic Pathways. Biometals.

[4] Johnson D.C., Dean D.R., Smith A.D., Johnson M.K. (2005). Structure, Function, and Formation of Biological Iron-Sulfur Clusters. Annu. Rev. Biochem..

[5] Golinelli-Cohen M.-P., Bouton C. (2017). Fe-S Proteins Acting as Redox Switch: New Key Actors of Cellular Adaptive Responses. Curr. Chem. Biol..

[6] Fuss J.O., Tsai C.-L., Ishida J.P., Tainer J.A. (2015). Emerging Critical Roles of Fe-S Clusters in DNA Replication and Repair. Biochim. Biophys. Acta.

[7] Colca J.R., McDonald W.G., Waldon D.J., Leone J.W., Lull J.M., Bannow C.A., Lund E.T., Mathews W.R. (2004). Identification of a Novel Mitochondrial Protein (“mitoNEET”) Cross-Linked Specifically by a Thiazolidinedione Photoprobe. Am. J. Physiol. Endocrinol. Metab..

[8] Lin J., Zhang L., Lai S., Ye K. (2011). Structure and Molecular Evolution of CDGSH Iron-Sulfur Domains. PLoS ONE.

[9] Mittler R., Darash-Yahana M., Sohn Y.S., Bai F., Song L., Cabantchik I.Z., Jennings P.A., Onuchic J.N., Nechushtai R. (2019). NEET Proteins: A New Link Between Iron Metabolism, Reactive Oxygen Species, and Cancer. Antioxid. Redox Signal..

[10] Nechushtai R., Karmi O., Zuo K., Marjault H.-B., Darash-Yahana M., Sohn Y.-S., King S.D., Zandalinas S.I., Carloni P., Mittler R. (2020). The Balancing Act of NEET Proteins: Iron, ROS, Calcium and Metabolism. Biochim. Biophys. Acta Mol. Cell Res..

[11] Wiley S.E., Murphy A.N., Ross S.A., van der Geer P., Dixon J.E. (2007). MitoNEET Is an Iron-Containing Outer Mitochondrial Membrane Protein That Regulates Oxidative Capacity. Proc. Natl. Acad. Sci. USA.

[12] Hou X., Liu R., Ross S., Smart E.J., Zhu H., Gong W. (2007). Crystallographic Studies of Human MitoNEET. J. Biol. Chem..

[13] Lin J., Zhou T., Ye K., Wang J. (2007). Crystal Structure of Human MitoNEET Reveals Distinct Groups of Iron–Sulfur Proteins. Proc. Natl. Acad. Sci. USA.

[14] Conlan A.R., Paddock M.L., Axelrod H.L., Cohen A.E., Abresch E.C., Wiley S., Roy M., Nechushtai R., Jennings P.A. (2009). The Novel 2Fe–2S Outer Mitochondrial Protein MitoNEET Displays Conformational Flexibility in Its N-Terminal Cytoplasmic Tethering Domain. Acta Crystallogr. Sect. F Struct. Biol. Cryst. Commun..

[15] Paddock M.L., Wiley S.E., Axelrod H.L., Cohen A.E., Roy M., Abresch E.C., Capraro D., Murphy A.N., Nechushtai R., Dixon J.E. (2007). MitoNEET Is a Uniquely Folded 2Fe 2S Outer Mitochondrial Membrane Protein Stabilized by Pioglitazone. Proc. Natl. Acad. Sci. USA.

[16] Baxter E.L., Jennings P.A., Onuchic J.N. (2011). Interdomain Communication Revealed in the Diabetes Drug Target MitoNEET. Proc. Natl. Acad. Sci. USA.

[17] Tamir S., Paddock M.L., Darash-Yahana-Baram M., Holt S.H., Sohn Y.S., Agranat L., Michaeli D., Stofleth J.T., Lipper C.H., Morcos F. (2015). Structure–Function Analysis of NEET Proteins Uncovers Their Role as Key Regulators of Iron and ROS Homeostasis in Health and Disease. Biochim. Biophys. Acta Mol. Cell Res..

[18] Ferecatu I., Gonçalves S., Golinelli-Cohen M.-P., Clémancey M., Martelli A., Riquier S., Guittet E., Latour J.-M., Puccio H., Drapier J.-C. (2014). The Diabetes Drug Target MitoNEET Governs a Novel Trafficking Pathway to Rebuild an Fe-S Cluster into Cytosolic Aconitase/Iron Regulatory Protein 1. J. Biol. Chem..

[19] Lipper C.H., Karmi O., Sohn Y.S., Darash-Yahana M., Lammert H., Song L., Liu A., Mittler R., Nechushtai R., Onuchic J.N. (2018). Structure of the Human Monomeric NEET Protein MiNT and Its Role in Regulating Iron and Reactive Oxygen Species in Cancer Cells. Proc. Natl. Acad. Sci. USA.

[20] Iwasaki T., Samoilova R.I., Kounosu A., Ohmori D., Dikanov S.A. (2009). Continuous-Wave and Pulsed EPR Characterization of the [2Fe-2S](Cys)3(His)1 Cluster in Rat MitoNEET. J. Am. Chem. Soc..

[21] Kusminski C.M., Holland W.L., Sun K., Park J., Spurgin S.B., Lin Y., Askew G.R., Simcox J.A., McClain D.A., Li C. (2012). MitoNEET-Driven Alterations in Adipocyte Mitochondrial Activity Reveal a Crucial Adaptive Process That Preserves Insulin Sensitivity in Obesity. Nat. Med..

[22] Lee S., Seok B.G., Lee S.-J., Chung S.W. (2022). Inhibition of MitoNEET Attenuates LPS-Induced Inflammation and Oxidative Stress. Cell Death Dis..

[23] Yonutas H.M., Hubbard W.B., Pandya J.D., Vekaria H.J., Geldenhuys W.J., Sullivan P.G. (2020). Bioenergetic Restoration and Neuroprotection after Therapeutic Targeting of MitoNEET: New Mechanism of Pioglitazone Following Traumatic Brain Injury. Exp. Neurol..

[24] Vernay A., Marchetti A., Sabra A., Jauslin T.N., Rosselin M., Scherer P.E., Demaurex N., Orci L., Cosson P. (2017). MitoNEET-Dependent Formation of Intermitochondrial Junctions. Proc. Natl. Acad. Sci. USA.

[25] Sohn Y.-S., Tamir S., Song L., Michaeli D., Matouk I., Conlan A.R., Harir Y., Holt S.H., Shulaev V., Paddock M.L. (2013). NAF-1 and MitoNEET Are Central to Human Breast Cancer Proliferation by Maintaining Mitochondrial Homeostasis and Promoting Tumor Growth. Proc. Natl. Acad. Sci. USA.

[26] Salem A.F., Whitaker-Menezes D., Howell A., Sotgia F., Lisanti M.P. (2012). Mitochondrial Biogenesis in Epithelial Cancer Cells Promotes Breast Cancer Tumor Growth and Confers Autophagy Resistance. Cell Cycle.

[27] Geldenhuys W.J., Piktel D., Moore J.C., Rellick S.L., Meadows E., Pinti M.V., Hollander J.M., Ammer A.G., Martin K.H., Gibson L.F. (2021). Loss of the Redox Mitochondrial Protein MitoNEET Leads to Mitochondrial Dysfunction in B-Cell Acute Lymphoblastic Leukemia. Free Radic. Biol. Med..

[28] Molino D., Pila-Castellanos I., Marjault H.-B., Dias Amoedo N., Kopp K., Rochin L., Karmi O., Sohn Y.-S., Lines L., Hamaï A. (2020). Chemical Targeting of NEET Proteins Reveals Their Function in Mitochondrial Morphodynamics. EMBO Rep..

[29] Lipper C.H., Stofleth J.T., Bai F., Sohn Y.-S., Roy S., Mittler R., Nechushtai R., Onuchic J.N., Jennings P.A. (2019). Redox-Dependent Gating of VDAC by MitoNEET. Proc. Natl. Acad. Sci. USA.

[30] Karmi O., Marjault H.-B., Bai F., Roy S., Sohn Y.-S., Darash Yahana M., Morcos F., Ioannidis K., Nahmias Y., Jennings P.A. (2022). A VDAC1-Mediated NEET Protein Chain Transfers [2Fe-2S] Clusters between the Mitochondria and the Cytosol and Impacts Mitochondrial Dynamics. Proc. Natl. Acad. Sci. USA.

[31] Kusminski C.M., Park J., Scherer P.E. (2014). MitoNEET-Mediated Effects on Browning of White Adipose Tissue. Nat. Commun..

[32] Moreno-Navarrete J.M., Moreno M., Ortega F., Sabater M., Xifra G., Ricart W., Fernández-Real J.M. (2016). CISD1 in Association with Obesity-Associated Dysfunctional Adipogenesis in Human Visceral Adipose Tissue. Obesity.

[33] Geldenhuys W.J., Benkovic S.A., Lin L., Yonutas H.M., Crish S.D., Sullivan P.G., Darvesh A.S., Brown C.M., Richardson J.R. (2017). MitoNEET (CISD1) Knockout Mice Show Signs of Striatal Mitochondrial Dysfunction and a Parkinson’s Disease Phenotype. ACS Chem. Neurosci..

[34] Lipper C.H., Paddock M.L., Onuchic J.N., Mittler R., Nechushtai R., Jennings P.A. (2015). Cancer-Related NEET Proteins Transfer 2Fe-2S Clusters to Anamorsin, a Protein Required for Cytosolic Iron-Sulfur Cluster Biogenesis. PLoS ONE.

[35] Zuris J.A., Harir Y., Conlan A.R., Shvartsman M., Michaeli D., Tamir S., Paddock M.L., Onuchic J.N., Mittler R., Cabantchik Z.I. (2011). Facile Transfer of [2Fe-2S] Clusters from the Diabetes Drug Target MitoNEET to an Apo-Acceptor Protein. Proc. Natl. Acad. Sci. USA.

[36] Landry A.P., Wang Y., Cheng Z., Crochet R.B., Lee Y.-H., Ding H. (2017). Flavin Nucleotides Act as Electron Shuttles Mediating Reduction of the [2Fe-2S] Clusters in Mitochondrial Outer Membrane Protein MitoNEET. Free Radic. Biol. Med..

[37] Wang Y., Landry A.P., Ding H. (2017). The Mitochondrial Outer Membrane Protein MitoNEET Is a Redox Enzyme Catalyzing Electron Transfer from FMNH2 to Oxygen or Ubiquinone. J. Biol. Chem..

[38] Tasnim H., Landry A.P., Fontenot C.R., Ding H. (2020). Exploring the FMN Binding Site in the Mitochondrial Outer Membrane Protein MitoNEET. Free Radic. Biol. Med..

[39] Landry A.P., Cheng Z., Ding H. (2015). Reduction of Mitochondrial Protein MitoNEET [2Fe-2S] Clusters by Human Glutathione Reductase. Free Radic. Biol. Med..

[40] Camponeschi F., Ciofi-Baffoni S., Banci L. (2017). Anamorsin/Ndor1 Complex Reduces [2Fe-2S]-MitoNEET via a Transient Protein-Protein Interaction. J. Am. Chem. Soc..

[41] Golinelli-Cohen M.-P., Lescop E., Mons C., Gonçalves S., Clémancey M., Santolini J., Guittet E., Blondin G., Latour J.-M., Bouton C. (2016). Redox Control of the Human Iron-Sulfur Repair Protein MitoNEET Activity via Its Iron-Sulfur Cluster. J. Biol. Chem..

[42] Bak D.W., Zuris J.A., Paddock M.L., Jennings P.A., Elliott S.J. (2009). Redox Characterization of the FeS Protein MitoNEET and Impact of Thiazolidinedione Drug Binding. Biochemistry.

[43] Tirrell T.F., Paddock M.L., Conlan A.R., Smoll E.J., Nechushtai R., Jennings P.A., Kim J.E. (2009). Resonance Raman Studies of the (His)(Cys)3 2Fe-2S Cluster of MitoNEET: Comparison to the (Cys)4 Mutant and Implications of the Effects of PH on the Labile Metal Center. Biochemistry.

[44] Landry A.P., Ding H. (2014). Redox Control of Human Mitochondrial Outer Membrane Protein MitoNEET [2Fe-2S] Clusters by Biological Thiols and Hydrogen Peroxide. J. Biol. Chem..

[45] Schröter T., Hatzfeld O.M., Gemeinhardt S., Korn M., Friedrich T., Ludwig B., Link T.A. (1998). Mutational Analysis of Residues Forming Hydrogen Bonds in the Rieske [2Fe-2S] Cluster of the Cytochrome Bc1 Complex in Paracoccus Denitrificans. Eur. J. Biochem..

[46] Bak D.W., Elliott S.J. (2013). Conserved Hydrogen Bonding Networks of MitoNEET Tune FeS Cluster Binding and Structural Stability. Biochemistry.

[47] Pesce L., Calandrini V., Marjault H.-B., Lipper C.H., Rossetti G., Mittler R., Jennings P.A., Bauer A., Nechushtai R., Carloni P. (2017). Molecular Dynamics Simulations of the [2Fe-2S] Cluster-Binding Domain of NEET Proteins Reveal Key Molecular Determinants That Induce Their Cluster Transfer/Release. J. Phys. Chem. B.

[48] Song G., Tian F., Liu H., Li G., Zheng P. (2021). Pioglitazone Inhibits Metal Cluster Transfer of MitoNEET by Stabilizing the Labile Fe-N Bond Revealed at Single-Bond Level. J. Phys. Chem. Lett..

[49] Zhou T., Lin J., Feng Y., Wang J. (2010). Binding of Reduced Nicotinamide Adenine Dinucleotide Phosphate Destabilizes the Iron−Sulfur Clusters of Human MitoNEET. Biochemistry.

[50] Wiley S.E., Paddock M.L., Abresch E.C., Gross L., van der Geer P., Nechushtai R., Murphy A.N., Jennings P.A., Dixon J.E. (2007). The Outer Mitochondrial Membrane Protein MitoNEET Contains a Novel Redox-Active 2Fe-2S Cluster. J. Biol. Chem..

[51] Noodleman L., Baerends E.J. (1984). Electronic Structure, Magnetic Properties, ESR, and Optical Spectra for 2-Iron Ferredoxin Models by LCAO-X.Alpha. Valence Bond Theory. J. Am. Chem. Soc..

[52] Karlsson A., Parales J.V., Parales R.E., Gibson D.T., Eklund H., Ramaswamy S. (2000). The Reduction of the Rieske Iron–Sulfur Cluster in Naphthalene Dioxygenase by X-Rays. J. Inorg. Biochem..

[53] Fujinaga J., Gaillard J., Meyer J. (1993). Mutated Forms of a [2Fe-2S] Ferredoxin with Serine Ligands to the Iron-Sulfur Cluster. Biochem. Biophys. Res. Commun..

[54] Meyer J., Fujinaga J., Gaillard J., Lutz M. (1994). Mutated Forms of the [2Fe-2S] Ferredoxin from Clostridium Pasteurianum with Noncysteinyl Ligands to the Iron-Sulfur Cluster. Biochemistry.

[55] Banci L., Ciofi-Baffoni S., Mikolajczyk M., Winkelmann J., Bill E., Pandelia M.-E. (2013). Human Anamorsin Binds [2Fe-2S] Clusters with Unique Electronic Properties. J. Biol. Inorg. Chem..

[56] Banci L., Brancaccio D., Ciofi-Baffoni S., Del Conte R., Gadepalli R., Mikolajczyk M., Neri S., Piccioli M., Winkelmann J. (2014). [2Fe-2S] Cluster Transfer in Iron-Sulfur Protein Biogenesis. Proc. Natl. Acad. Sci. USA.

[57] Cai K., Tonelli M., Frederick R.O., Markley J.L. (2017). Human Mitochondrial Ferredoxin 1 (FDX1) and Ferredoxin 2 (FDX2) Both Bind Cysteine Desulfurase and Donate Electrons for Iron-Sulfur Cluster Biosynthesis. Biochemistry.

[58] Heidrich H.-G., Albracht S.P.J., Bäckström D. (1978). Two Iron—Sulfur Centers in Mitochondrial Outer Membranes from Beef Heart as Prepared by Free-Flow Electrophoresis. FEBS Lett..

[59] Dicus M.M., Conlan A., Nechushtai R., Jennings P.A., Paddock M.L., Britt R.D., Stoll S. (2010). Binding of Histidine in the (Cys)3(His)1-Coordinated [2Fe−2S] Cluster of Human MitoNEET. J. Am. Chem. Soc..

[60] Guigliarelli B., Bertrand P., Sykes A.G. (1999). Application of EPR Spectroscopy to the Structural and Functional Study of Iron-Sulfur Proteins. Advances in Inorganic Chemistry.

[61] Bertrand P., More C., Guigliarelli B., Fournel A., Bennett B., Howes B. (1994). Biological Polynuclear Clusters Coupled by Magnetic Interactions: From the Point Dipole Approximation to a Local Spin Model. J. Am. Chem. Soc..

[62] Cline J.F., Hoffman B.M., Mims W.B., LaHaie E., Ballou D.P., Fee J.A. (1985). Evidence for N Coordination to Fe in the [2Fe-2S] Clusters of Thermus Rieske Protein and Phthalate Dioxygenase from Pseudomonas. J. Biol. Chem..

[63] Gurbiel R.J., Batie C.J., Sivaraja M., True A.E., Fee J.A., Hoffman B.M., Ballou D.P. (1989). Electron-Nuclear Double Resonance Spectroscopy of 15N-Enriched Phthalate Dioxygenase from Pseudomonas Cepacia Proves That Two Histidines Are Coordinated to the [2Fe-2S] Rieske-Type Clusters. Biochemistry.

[64] Iwata S., Saynovits M., Link T.A., Michel H. (1996). Structure of a Water Soluble Fragment of the “Rieske” Iron-Sulfur Protein of the Bovine Heart Mitochondrial Cytochrome Bc1 Complex Determined by MAD Phasing at 1.5 A Resolution. Structure.

[65] Pandelia M.-E., Lanz N.D., Booker S.J., Krebs C. (2015). Mössbauer Spectroscopy of Fe/S Proteins. Biochim. Biophys. Acta-Mol. Cell Res..

[66] Fee J.A., Findling K.L., Yoshida T., Hille R., Tarr G.E., Hearshen D.O., Dunham W.R., Day E.P., Kent T.A., Münck E. (1984). Purification and Characterization of the Rieske Iron-Sulfur Protein from Thermus Thermophilus. Evidence for a [2Fe-2S] Cluster Having Non-Cysteine Ligands. J. Biol. Chem..

[67] Fleischhacker A.S., Stubna A., Hsueh K.-L., Guo Y., Teter S.J., Rose J.C., Brunold T.C., Markley J.L., Münck E., Kiley P.J. (2012). Characterization of the [2Fe-2S] Cluster of *Escherichia coli* Transcription Factor IscR. Biochemistry.

[68] Chandramouli K., Unciuleac M.-C., Naik S., Dean D.R., Huynh B.H., Johnson M.K. (2007). Formation and Properties of [4Fe-4S] Clusters on the IscU Scaffold Protein. Biochemistry.

[69] Münck E., Debrunner P.G., Tsibris J.C., Gunsalus I.C. (1972). Mössbauer Parameters of Putidaredoxin and Its Selenium Analog. Biochemistry.

[70] Meyer J., Clay M.D., Johnson M.K., Stubna A., Münck E., Higgins C., Wittung-Stafshede P. (2002). A Hyperthermophilic Plant-Type [2Fe-2S] Ferredoxin from Aquifex Aeolicus Is Stabilized by a Disulfide Bond. Biochemistry.

[71] Wolfe M.D., Altier D.J., Stubna A., Popescu C.V., Münck E., Lipscomb J.D. (2002). Benzoate 1,2-Dioxygenase from Pseudomonas Putida: Single Turnover Kinetics and Regulation of a Two-Component Rieske Dioxygenase. Biochemistry.

[72] Garcia-Serres R., Clémancey M., Latour J.-M., Blondin G. (2018). Contribution of Mössbauer Spectroscopy to the Investigation of Fe/S Biogenesis. J. Biol. Inorg. Chem..

[73] Li H., Mapolelo D.T., Dingra N.N., Naik S.G., Lees N.S., Hoffman B.M., Riggs-Gelasco P.J., Huynh B.H., Johnson M.K., Outten C.E. (2009). The Yeast Iron Regulatory Proteins Grx3/4 and Fra2 Form Heterodimeric Complexes Containing a [2Fe-2S] Cluster with Cysteinyl and Histidyl Ligation. Biochemistry.

[74] Kristina Beilschmidt L., Choudens S., Fournier M., Sanakis Y., Hograindleur M.-A., Clémancey M., Blondin G., Schmucker S., Eisenmann A., Weiss A. (2017). ISCA1 Is Essential for Mitochondrial Fe4S4 Biogenesis in Vivo. Nat. Comm..

[75] Camponeschi F., Gallo A., Piccioli M., Banci L. (2021). The Long-Standing Relationship between Paramagnetic NMR and Iron–Sulfur Proteins: The MitoNEET Example. An Old Method for New Stories or the Other Way Around?. Magn. Reson..

[76] Banci L., Camponeschi F., Ciofi-Baffoni S., Piccioli M. (2018). The NMR Contribution to Protein-Protein Networking in Fe-S Protein Maturation. J. Biol. Inorg. Chem..

[77] Piccioli M. (2020). Paramagnetic NMR Spectroscopy Is a Tool to Address Reactivity, Structure, and Protein–Protein Interactions of Metalloproteins: The Case of Iron–Sulfur Proteins. Magnetochemistry.

[78] Trindade I.B., Coelho A., Cantini F., Piccioli M., Louro R.O. (2022). NMR of Paramagnetic Metalloproteins in Solution: Ubi Venire, Quo Vadis?. J. Inorg. Biochem..

[79] Trindade I.B., Invernici M., Cantini F., Louro R.O., Piccioli M. (2021). PRE-Driven Protein NMR Structures: An Alternative Approach in Highly Paramagnetic Systems. FEBS J..

[80] Invernici M., Selvolini G., Silva J.M., Marrazza G., Ciofi-Baffoni S., Piccioli M. (2022). Interconversion between [2Fe–2S] and [4Fe–4S] Cluster Glutathione Complexes. Chem. Commun..

[81] Brancaccio D., Gallo A., Piccioli M., Novellino E., Ciofi-Baffoni S., Banci L. (2017). [4Fe-4S] Cluster Assembly in Mitochondria and Its Impairment by Copper. J. Am. Chem. Soc..

[82] Camponeschi F., Prusty N.R., Heider S.A.E., Ciofi-Baffoni S., Banci L. (2020). GLRX3 Acts as a [2Fe–2S] Cluster Chaperone in the Cytosolic Iron–Sulfur Assembly Machinery Transferring [2Fe–2S] Clusters to NUBP1. J. Am. Chem. Soc..

[83] Brancaccio D., Gallo A., Mikolajczyk M., Zovo K., Palumaa P., Novellino E., Piccioli M., Ciofi-Baffoni S., Banci L. (2014). Formation of [4Fe-4S] Clusters in the Mitochondrial Iron-Sulfur Cluster Assembly Machinery. J. Am. Chem. Soc..

[84] Camponeschi F., Muzzioli R., Ciofi-Baffoni S., Piccioli M., Banci L. (2019). Paramagnetic 1H NMR Spectroscopy to Investigate the Catalytic Mechanism of Radical S-Adenosylmethionine Enzymes. J. Mol. Biol..

[85] Banci L., Bertini I., Luchinat C. (1990). The ^1^H NMR Parameters of Magnetically Coupled Dimers—The Fe_2_S_2_ Proteins as an Example. Struct. Bond..

[86] Holz R.C., Small F.J., Ensign S.A. (1997). Proton Nuclear Magnetic Resonance Investigation of the [2Fe-2S](1-)-Containing “Rieske-Type” Protein from Xanthobacter Strain Py2. Biochemistry.

[87] Skjeldal L., Markley J.L., Coghlan V.M., Vickery L.E. (1991). 1H NMR Spectra of Vertebrate [2Fe-2S] Ferredoxins. Hyperfine Resonances Suggest Different Electron Delocalization Patterns from Plant Ferredoxins. Biochemistry.

[88] Maio N., Rouault T.A. (2020). Outlining the Complex Pathway of Mammalian Fe-S Cluster Biogenesis. Trends Biochem. Sci..

[89] Machonkin T.E., Westler W.M., Markley J.L. (2004). Strategy for the Study of Paramagnetic Proteins with Slow Electronic Relaxation Rates by NMR Spectroscopy: Application to Oxidized Human [2Fe-2S] Ferredoxin. J. Am. Chem. Soc..

[90] Xia B., Jenk D., LeMaster D.M., Westler W.M., Markley J.L. (2000). Electron-Nuclear Interactions in Two Prototypical [2Fe-2S] Proteins: Selective (Chiral) Deuteration and Analysis of (1)H and (2)H NMR Signals from the Alpha and Beta Hydrogens of Cysteinyl Residues That Ligate the Iron in the Active Sites of Human Ferredoxin and Anabaena 7120 Vegetative Ferredoxin. Arch. Biochem. Biophys..

[91] Banci L., Bertini I., Ciofi-Baffoni S., Boscaro F., Chatzi A., Mikolajczyk M., Tokatlidis K., Winkelmann J. (2011). Anamorsin Is a [2Fe-2S] Cluster-Containing Substrate of the Mia40-Dependent Mitochondrial Protein Trapping Machinery. Chem. Biol..

[92] Spronk C.A.E.M., Żerko S., Górka M., Koźmiński W., Bardiaux B., Zambelli B., Musiani F., Piccioli M., Basak P., Blum F.C. (2018). Structure and Dynamics of Helicobacter Pylori Nickel-Chaperone HypA: An Integrated Approach Using NMR Spectroscopy, Functional Assays and Computational Tools. J. Biol. Inorg. Chem..

[93] Cheng H., Xia B., Reed G.H., Markley J.L. (1994). Optical, EPR, and 1H NMR Spectroscopy of Serine-Ligated [2Fe-2S] Ferredoxins Produced by Site-Directed Mutagenesis of Cysteine Residues in Recombinant Anabaena 7120 Vegetative Ferredoxin. Biochemistry.

[94] Trindade I.B., Hernandez G., Lebègue E., Barrière F., Cordeiro T., Piccioli M., Louro R.O. (2021). Conjuring up a Ghost: FhuF—A Ferric-Siderophore Reductase of Unknown Structure. J. Biol. Inorg. Chem..

[95] Dugad L.B., La Mar G.N., Banci L., Bertini I. (1990). Identification of Localized Redox States in Plant-Type Two-Iron Ferredoxins Using the Nuclear Overhauser Effect. Biochemistry.

[96] Bertini I., Capozzi F., Luchinat C., Piccioli M., Vila A.J. (1994). The Fe4S4 Centers in Ferredoxins Studied through Proton and Carbon Hyperfine Coupling. Sequence-Specific Assignments of Cysteines in Ferredoxins from Clostridium Acidi Urici and Clostridium Pasteurianum. J. Am. Chem. Soc..

[97] Holt S.H., Darash-Yahana M., Sohn Y.S., Song L., Karmi O., Tamir S., Michaeli D., Luo Y., Paddock M.L., Jennings P.A. (2016). Activation of Apoptosis in NAF-1-Deficient Human Epithelial Breast Cancer Cells. J. Cell Sci..

[98] Darash-Yahana M., Pozniak Y., Lu M., Sohn Y.-S., Karmi O., Tamir S., Bai F., Song L., Jennings P.A., Pikarsky E. (2016). Breast Cancer Tumorigenicity Is Dependent on High Expression Levels of NAF-1 and the Lability of Its Fe-S Clusters. Proc. Natl. Acad. Sci. USA.

